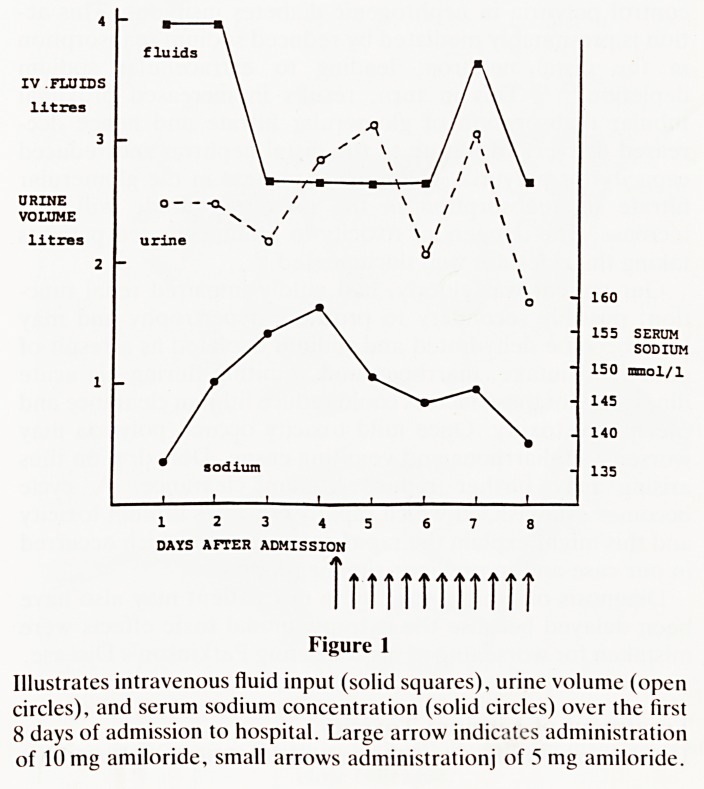# Saline Infusion and Amiloride in the Management of Lithium Toxicity

**Published:** 1990-09

**Authors:** Ian W D Czerniewski, Jacquline A Short, A A McConnell

## Abstract

This paper describes the case of a 74 year old patient who became lithium toxic after 15 years of lithium therapy. We discuss the clinical presentation of the case and some of the possible causes of the sudden development of his toxicity. Although haemodialysis is the treatment of choice for severe lithium toxicity it is not always available. In this paper we propose that the combination of saline diuresis and Amiloride may provide a suitable alternative in the management of lithium toxicity.


### Saline Infusion and Amiloride in the Management of Lithium Toxicity

**Ian W D Czerniewski**
**Jacquline A Short**
**A A McConnell**

### SUMMARY

**This paper describes the case of a 74 year old patient who became lithium toxic after 15 years of lithium therapy. We discuss the clinical presentation of the case and some of the possible causes of the sudden development of his toxicity. Although haemodialysis is the treatment of choice for severe lithium toxicity it is not always available. In this paper we propose that the combination of saline diuresis and Amiloride may provide a suitable alternative in the management of lithium toxicity.**

### INTRODUCTION

Lithium carbonate is widely used in the treatment of manic depressive illness. Lithium has a narrow therapeutic index and toxicity is well recognised.[1,2,3] We report a case of lithium toxicity, discuss some aspects of the condition and propose a modification in its management.

### CASE REPORT

A 74 year old man was admitted to a geriatric unit as an emergency. He had been treated with lithium carbonate, 800 mg daily, for 15 years for manic depression. Regular serum lithium estimations had consistently been within the local therepeutic range (0.4—1.2 mmol/L). For two years he had reported mild short term amnesia and suffered from mild tremor and polyuria. Five months prior to admission mild rigidity, bradykinesia and worsening tremor were noted. Serum lithium level then was 1.0 mmol/L. A neurologist diagnosed Parkinson's Disease and prescribed Madopar (Roche) 125 mg initially three times daily then five times daily. The extrapyramidal features improved.

Ten days prior to admission the patient suffered an episode of acute urinary retention which resolved spontaneously. At that time, increasing tremor and rigidity were noted once more and Selegeline, 5 mg daily, was added to his anti-Parkinsonian treatment. Over the ten days prior to admission, he became increasingly immobile, rigid and tremulous. Ataxia, dysarthria, disorientation, confusion and urinary incontinence developed and worsened and he suffered severe vomiting and diarrhoea. Despite these features all medication was continued throughout the illness. There was nothing to suggest deliberate overdosage.

On admission he was bed bound, grunting, barely responsive and grossly dehydrated with poor skin turgor, pulse rate 110 beats per minute and blood pressure 110/50 mm Hg. Muscular rigidity with firm extension of the limbs, coarse tremor, cogwheeling, hyperreflexia and gross myclonic jaw jerks were observed. The only other significant finding was of a smooth enlarged prostate.

A clinical diagnosis of lithium toxicity was made and confirmed by a serum level of 3.9 mmol/L. Other notable serum levels were: Urea 36.1 mmol/L.

All existing therapy was discontinued. Haemodialysis facilities were denied and intravenous rehydration was commenced. Four litres of 0.9% saline solution and four litres of 5% glucose solution were given over 24 hours. In this peiod 5 litres of dilute urine were passed per catheter. Serum sodium rose to 148 mmol/L and subsequent rehydration was with 5% glucose solution alone. Despite persistent clinical dehydration he continued to pass copious and dilute urine (urine osmolality 163 mmol/kg) and serum sodium rose to 159 mmol/L after 4 days. (Figure 1).

A single dose of Amiloride 10 mg was given on day 5 and further eight-hourly doses of Amiloride 5 mg were given until day 11. Over this period serum sodium and urea returned to normal, hydration improved and urine output fell below fluid input. Serum lithium fell to 1.0 mmol/L by day 6 and 09.2 mmol/L by day 14.

Diarrhoa and vomiting settled over the first week. There was little neurological change over the first 5 days, then stupor gave way to confusion with amnesia, dysarthria and gross ataxia which gradually resolved. Rigidity, cogwheeling and tremor settled during the second week and reached a steady state. After one month Madopar, 125 mg three times daily, was recommenced with successful control of extrapyramidal features.

The polyuria settled and after one month renal function reached a steady level with serum urea 10.1 mmol/L and serum creatinine 141 umol/L.

The only residual abnormalities were impaired short term memory and mild ataxia which was still improving after three months.

### DISCUSSION

Our patient exhibited many of the side effects and then toxic effects of lithium.

**Figure 1**

Illustrates intravenous fluid input (solid squares), urine volume (open circles), and serum sodium concentration (solid circles) over the first 8 days of admission to hospital. Large arrow indicates administration of 10 mg amiloride, small arrows administrationj of 5 mg amiloride.

Tremor, fatigue and polyuria are common side effects of lithium occurring at thereapeutic levels.[4] Gross tremor, dysarthria, ataxia, confusion extrapyramidal features, diarrhoea and vomiting are well recognised in lithium toxicity. Severe poisoning may lead to stupor, coma and death.[3,5,6] The persistence of neurological side effects for several weeks after peak serum lithium levels, presumably related to slow release of lithium from nerve tissue has also been described, although permanent neurological sequelae may also occur after lithium toxicity.[5]

### Causes of Toxicity

Lithium toxicity may arise from excessive ingestion or from reduced excretion of lithium.

Lithium is excreted by the kidneys and elderly patients[7] and those with impaired renal function[8] are known to have reduced lithium clearance.

Dehydration, presumably because of reduced glomerular filtration, also leads to reduced lithium clearance.[9] Dehydration may occur in lithium treated patients who do not maintain an adequate fluid input since lithium itself causes polyuria.[10] This polyuria is most commonly caused by the direct action of lithium on renal collecting duct epithelial cells.[11] Lithium interferes with the action of antidiuretic hormone (ADH) thus causing a nephrogenic diabetes insipidus.[10] This effect has been ameliorated by the administration of Amiloride to patients on long term lithium therapy[12], and it is thought that Amiloride directly blocks the action of lithium on the collecting duct. Rarely lithium may also affect pituitary ADH secretion[10] (central diabetes insipidus) or may induce a permanent interstitial nephropathy[13] and both of these effects may aggravate polyuria.

Sodium depletion also causes reduced lithium clearance.[14] While sodium and lithium appear to be reabsorbed by a common mechanism in the renal proximal tubule, lithium, unlike sodium, appears not to be significantly reabsorbed (or secreted) in the remainder of the nephron.[14, 15] Sodium depletion may arise from inadequate intake of sodium, from severe gastrointestinal disturbances or from the use of diuretics, particularly thiazide diuretics.[16] Consequently proximal tubular reabsorption of sodium (and lithium) is increased and lithium toxicity may ensue. The role of thiazides is of particular interest. Thiazide diuretics may paradoxically be used to control polyuria in nephrogenic diabetes insipidus. This action is presumably mediated by reduced sodium reabsorption in the distal nephron, leading to extracellular sodium depletion.[12, 16] This in turn, results in increased proximal tubular reabsorption of glomerular filtrate and hence decreased delivery of filtrate to the distal nephron and reduced capacity for polyuria. If lithium is present in the glomerular filtrate its reabsorption in the proximal tubule will also increase. The dangers of toxicity in lithium treated patients taking thiazides are well documented.[16]

Our patient was elderly, had mildly impaired renal function, possibly secondary to prostatic hypertrophy and may have become dehydrated and sodium depleted as a result of poor fluid intake, diarrhoea and vomiting during his acute illness. All of these factors could reduce lithium clearance and precipitate toxiity. Once mild toxicity occurs, polyuria may worsen and diarrhoea and vomiting ensue. Dehydration thus arising may further reduce lithium clearance. A cycle becomes establish3ed which rapidly escalates lithium toxicity and this might explain the rapid deterioration which occurred in our case and in previous similar reports.[1,2]

Diagnosis of lithium toxicity in our patient may also have been delayed because the extrapyramidal toxic effects were mistaken for worsening of his coexisting Parkinson's Disease.

### Treatment of Lithium Toxicity

There is no antidote to lithium and treatment of toxicity is directed at support and active removal of lithium. In severe toxicity this is best achieved by haemodialysis if the facility is available.[2, 6]

Since severely toxic patients are often dehydrated and since the correction of sodium depletion is known to improve lithium clearance,[14] the administration of saline infusion is a logical step in the management of lithium toxicity in the absence of haemodialysis. Saline infusion has indeed been used in previous reports[1,2] but caution is advised because electrolyte imbalance, particularly hypernatraemia may arise.[1,2] When this occurred in these reports, thiazides were given to control this complication, although we now question the logic of this.

Persistent dehydration and polyuria were the main problems during the early phase of our patient's admission. The hypernatraemia which developed probably resulted from the persistent polyuria resulting in a state of hypernatraemic dehydration.

As explained above, sodium depletion must occur before thiazides can significantly ameliorate polyuria in nephrogenic diabetes insipidus. If this occurs, lithium clearance will actually be reduced—an undesirable effect in the presence of lithium toxicity.

Amiloride has been used to ameliorate the polyuria of long-term lithium therapy,[12] but it has been suggested that this effect of Amiloride takes several days to develop.[12] There has been no report of the possible benefits of Amiloride during acute lithium intoxication. Since Amiloride specifically reduces lithium-induced polyuria as well as being mildly natriuretic, it is logical to administer the drug to acutely toxic patients who are polyuric and hypernatraemic. Amiloride does not reduce lithium clearance[12] and is therefore theoretically superior to thiazides in this situation.

Our patient was given only 4 litres of infused saline but became hypernatraemic and remained moderately polyuric. In previous cases more severe polyuria and hypernatraemia were encountered and after thiazide therapy there was a slow improvement in all parameters.[1,2] In our case the administration of Amiloride was rapidly followed by biochemcial and clinical improvement. This may have been coincidental. Nevertheless, we propose that evaluation is needed of the possible beneficial effects of Amiloride adminstration at the commencement of saline infusion in treating lithium toxicity. Polyuria may thus be reduced and the complication of severe hypernatraemia may be ameliorated or possibly abolished.

### ACKNOWLEDGEMENTS

Dr William Calwell (Consultant Physican) was directly involved in the care of this patient. He died suddenly, shortly afterwards, in November 1989. This paper is dedicated to his memory.

We wish to thank Dr G R Burston (Consultant Physican in charge of our patient) and Dr R M Hawley (Consultant Psychiatrist) for their help and encouragement in the preparation of this paper, and Mrs Mandy Cottle and Mr John Clegg for preparation of the typescript.

### REFERENCES

1. HANSEN, H. E., AMDISEN, A. (1978) Lithium Intoxication. *Quart. J. of Medicine* New series XLVII. 166 pp 123–144.
2. SIMARD, M., GUMBINER, B., LEE, A., LEIS, H., NORMAN, D. (1989) Lithium Carbonate Intoxication. *Arch Intern. Med.* **149**, 34–46
3. SCHOU, M., AMDISEN, A., TRAP-JENSEN, J. (1968) Lithium Poisoning *Am. J. Psychiatry* **125**, 520–527.
4. BONE, S., ROOSE, S. P., DUNNER, D. L., *et al.* (1980) Incidence of side effects in patients on long term Lithium therapy. *Am. J. Psychiatry* **137**, 103–104.
5. NEWMAN, P. K., SAUNDERS, M. (1979) Lithium Neurotoxicity. *Postgraduate Med. J.* **55** 701–703.

*Continued on p. 76*

**Figure 3**

A coronal slice through the anterior aspect of the temporal horn and the hippocampus. Inversion Recovery sequence. Normal control.

**Figure 4**

*Continued from p. 86*

SALINE INFUSION AND AMILORIDE IN THE TREATMENT OF LITHIUM TOXICITY.

6. SHOU, M. (1980) The Recognition and Management of Lithium Intoxication. In: *Handbook of Lithium Therapy Ed.* Johnson, F. N. Lancaster, England. MPT Press.
7. CHAPRON, D. J., CAMERON, I. R., WHITE, L. B., MERRAL, P. (1982) Observations on lithium disposition in the elderly. *J. Am. Geriatr. Soc.* **30**, 651–655.
8. DAVIS, J. M., FANN, W. E. (1971) Lithium. *Rev. Pharmacol* **11**, 285–302.
9. THOMSEN, K., OLESEN, O. V. (1979) The effect of water deprivation on lithium clearance and lithium excretion fraction in lithium-polyuric rats. *J. Pharmacol. Exp. Ther.* **209**, 327–329.
10. FORREST, J. N. Jr., COHEN, A. D., TORETTI, J., HIMMELHOCH, J. M., EPSTEIN, F. H. (1974) On the mechanism of Lithium induced diabete insipidus in man and the rat. *J. Clin. Invest.* **53**, 1115–1123.
11. BAYLIS, P. H., HEATH, D. A. (1978) Water disturbances in patients treated with oral lithium Carbonate. *Ann. Int. Med.* **88**, 607–609.
12. BATLLE, D. C., VON RIOTTE, A. B., GAVIRIA, M., GRUPP, M. (1985) Amelioration of polyuria by Amiloride in patients receiving long term Lithium therapy. *N. Engl. J. Med.* **312**, 408–414.
13. HANSEN, H. E., HESTBACH, J., SORENSEN, J. L., NORGAARD, K., HEILSKOV, J., AMDISEN, A. (1979) Chronic interstitial nephropathy in patients on long term Lithium treatment. *Quart. J. Med.* **48**, 577–591.
14. THOMSEN, K., SCHOU, M. (1968) Renal lithium excretion in Man. *Am. J. Physiol* **215**, 823–827.
15. THOMSEN, K. (1984) Lithium clearance: A new method for determining proximal and distal tubular reabsorption of sodium and water. *Nephron* **37**, 217–223.
16 PETERSEN, V., HVIDT, S., THOMSEN K., SCHOU, M. (1974) Effect of prolonged thiazide treatment on renal lithium clearance. *BMJ* **3**, 143–145.

## Figures and Tables

**Figure 1 f1:**